# 

**Published:** 1823-09-01

**Authors:** 


### BIBLIOGRAPHICAL RECORD;

OR,

*Books received for Review since the last Quarter.*

---

1. A Treatise on Materia Medica and Therapeutics. By JOHN EBERLE, M.D. Editor of the American Medical Recorder, &c. &c. Two volumes, 8vo, pp. 440 and pp. 537. Philadelphia, 1822.

---

2. Enchiridion:—or, a Hand for the One-handed. By GEORGE WEBB DERENZY, Captain, H.P. 82d Regiment. Octavo, pp. 60, with numerous wood-cuts. London, 1823, price 5s. boards.

☞ *To the thousands who are subjected by the chance of war, the hazards of machinery, the effect of accidents, disease, or malformation, to a loss of manual power on one side, Captain Derenzy's apparatus will prove an invaluable convenience and comfort, as well as a source of independence. No man who is maimed in the manner in question should hesitate to convince himself, by an inspection of the apparatus or a perusal of the work, whether or not we have over-estimated its advantages. The whole of the items amount only to* 10*l. and any of them may be got separately.*

---

3. The American Medical Recorder of original Papers and Intelligence in Medicine and Surgery, No. 21. Philadelphia, January 1823.

---

4. De la Douleur, considerée sous le Point de Vue de son Utilité en Medecine, et dans ses Rapports avec la Physiologie, l'Hygiéne, la Pathologie, et la Therapeutique. Par JACQ. ALEX. SALGUES, D.M. &c. 8vo, pp. 128. Paris, 1823.

---

5. An Essay on the baleful Influence of so frequently Washing Decks in His Majesty's Ships, on the Health of British Seamen: with Observations on the Prevention of Dry Rot in the Royal Navy. By ROBERT FINLAYSON, M.D. Member of the Royal College of Surgeons of London, and Surgeon in the Royal Navy. Octavo, pp. 85, with a Plate. London, 1823.

☞ *This little Essay is written with great spirit and intelligence. Although we are not disposed to go the whole length with Dr. Finlayson, respecting the baleful influence of moisture on the health of seamen, yet we are convinced that if the executive officers of ships were to hearken to Dr. F's instructions on very many points, the men under their charge would experience an increase of comfort and a dimunition of sickness.*

---

6. An Outline of Hints for the Political Organization and Moral Training of the Human Race: submitted, with deference, to the consideration of those who frame Laws for the Civil Government of Man, and more especially for those who direct, or profess to direct, Man to the true Worship of the Deity. By ROBERT JACKSON, M.D. Octavo, pp. 253. Price 9s. boards. London, July 1823.

☞ *The second chapter of this work contains the "Outline of a Plan of National Medical Establishment," which will, of course, deserve the attention of the profession. Any thing from the pen of Dr. Jackson ensures respect. We hope, in our next, to give some account of the Work.*

---

7 Sketches in Bedlam, or characteristic Traits of Insanity, as displayed in the Cases of 140 Patients of both Sexes, now, or recently, confined in New Bethlem, including Margaret Nicholson, James Hatfield, Patrick Walsh, Bannister Truelock, and many other extraordinary Maniacs, who have been transferred from Old Bethlem. To which are added, a succinct History of the Establishment, its Rules, Regulations, Forms of Admission, Treatment of Patients, &c By a Constant Observer. Octavo, pp. 312. 1823.

☞ *The above Work contains many curious particulars of insane persons, which are far from uninteresting to the profession. We shall, therefore, dedicate an article to this subject in our next number.*

---

8. A Treatise on Mental Derangement. Containing the Substance of the Gulstonian Lectures for May 1822. By Francis Willis, M. D. Fellow of the Royal College of Physicians. Octavo, pp 234. London. Longman and Co. 1823.

---

9. American Medical Recorder, No. 22, for April 1823. In exchange for Medico-Chirurgical Review.

---

10. The Philadelphia Journal of the Medical and Physical Sciences, No. 11, May 1823. In exchange for Medico-Chirurgical Review.

---

11. Transactions of the Associated Apothecaries and Surgeon-Apothecaries. Vol. I. Burgess and Hill, Sept. 1, 1823.

☞ *A copious review of this stranger in our next number.*

---

12. Lectures on the Operative Surgery of the Eye; being the Substance of that Part of the Course of Lectures on the Principles and Practice of Surgery which relates to the Diseases of that Organ; published for the Purpose of assisting in bringing the Management of these Complaints within the Principles which regulate the Practice of Surgery in general. By G. J. Guthrie, Deputy Inspector of Hospitals, &c. &c. Octavo, pp. 514. London, August 1823.

---

13. Prison Labour, &c. Correspondence and Communications addressed to His Majesty's Principal Secretary of State for the Home Department, concerning the Introduction of Tread-Mills into Prisons, with other Matters connected with the Subject of Prison Discipline. By Sir John Cox Hippesley, Bart. Bencher of the Inner Temple, (transmitted by the Author.) Octovo, pp. 228. London, August 1823.

☞ *We beg to return the worthy and able Baronet our thanks for the present of his interesting work on a curious and highly interesting subject —the employment of culprits. We shall take an early opportunity of examining the Work with care, as we see it embraces a question of Physiology of Hygiene, which falls properly in our line of study.*

---

14. The Life of William Hey, Esq. F.R.S. &c. &c. &c. By John Pearson, F.R.S. F.L.S. M.R.I.A. In two volumes, 8vo. *Second Edit.* 1823.

### XIII.

### TO CORRESPONDENTS.

☞ The principal Editor of this Journal being absent on a tour on the Continent at the time the present number was closed, several small articles of correspondence and intelligence must necessarily lie over till his return, when they will be attended to, no doubt.

---

The severe satire of "Infra Dig" could not be inserted in the superintending Editor's absence. The Editor, *ad interim*, however, cannot help expressing his surprise that the Committee at Somerset House should have admitted two such baboon-looking faces as those of Sir Charlatan Drawcausin and Son, over the portal of their highest room. Nor is his surprise less, that a metropolitan physician should *exhibit* himself at full length, on the walls of the same room, along-side of a human skeleton with a *crooked spine!* What is this but an *intra*, instead of an *extra-mural* advertisement, a la Bonassus, Eady, or Warren's Blacking? Bear this in mind, ye Censors of Warwick Lane!

---

The requests respecting the first volume of Mr. Clarke's Work on Female Discharges shall be complied with in our next number.

---

To the inquiry respecting Burns' Surgical Anatomy of the Neck, we can only answer that the work is exceedingly scarce, nor do we hear of any preparation for re-publishing it.

---

Letters, books for review, information, subscribers' names, &c. &c. are to be addressed to the Editor's residence in London, as usual:—he will return before the December number is published.

---

☞ We have given, in this number of the Journal, upwards of 20 pages of latter-press beyond the established limits.

### *Additional Subscribers since last Quarter.*

Abbott, Mr. William, (Student)
Adams, George, Esq. Surg. Colebrook, near Windsor
Atkinson, Thomas, Esq. Surgeon, Kilhan, Yorkshire
Bacon, George, Esq. To East Indies
Backhouse, Mr. Thos West Lodge, near Darlington
Barry, Mr. Surgeon, Jermyn-street, St. James's
Bates, Mr. Surgeon, Fleet Market
Bell, Mr. Oxford Street
Birdsall, Mr. Wm. Surg. Pickering
Bowen, Mr. Assistant Surgeon, His Majesty's Ship Isis (flag)—To Jamaica
Calvert, Dr. Phys. to the Forces, Edingly, Nottinghamshire
Chapman, Mr. York
Cheetham, Mr. Surgeon, Ashton-under-Lyne.
Christie, Mr. from Hon. East India Comp. Service, to North Brixton
Clarke, Mr. John, Surgeon, R.N.
Davidson, Mr. Thomas, Surgeon, Bredsen, Derby
Davies, Dr. Owen
Davies, Mr. Surgeon, Kensington
Donnett, Dr. Gibraltar
Denton, Mr. Surgeon, &c. Castle-street, Oxford-street
Drummond, Jas. L. M.D. Belfast
Elletson, Mr. John, Surg. Howden
Grace, Charles, M.D. Cupar, Fife
Grahame, Mr. William, East India Company's Service
Green, J. H. Esq Surgeon to St. Thomas's Hospital, Lincoln's-Inn-Fields
Goldwire, Dr. J. Memb. of the Royal College of Physicians—to Italy
Hall, Dr. Marshall, Nottingham
Hall, Mr. John, Assist. Surg R.N.
Hall, John, Assist. Surg. R.N. and Member Royal College Surgeons, London
Hall, W. J. Esq. Leeds
Hallion, J. W. Esq. Surgeon, 6, Upper Conway-street, Fitzroy Square
Harper, Rowland, Esq. Member of the Royal College of Surgeons, London, Etivale, Derbyshire
Hutchinson, Benj. Esq. Surgeon, Southwell
Hulton, Mr. Surg. Stayly Bridge, near Ashton-under-Lyne
Ingham, Dr. 22d Regiment
Jones, Dr. Edward, Paris
Kay, Mr. Surgeon, Ashton-under-Lyne
Long, Richard, Esq. M. D. Surg. to the Arthur's-town Dispensary, Wexford, Ireland
Ludlow, Samuel, Esq. Resident Surgeon, Delhi
Matthews, T. L. East India Company's Service
Mitchell, Mr. Surgeon, R.N.
Mountjoy, Mr. Fellow of the Royal College of Surgeons, Wotton under Edge
O'Grady, Mr. Apothecary to the Embassy, Paris
Percivall, William, Vet. Surgeon, Woolwich
Phillips, John, Esq. Pall Mall
Pierce, Mr. Wyniatt-street, Northampton Square, London
Poole, Mr. Robert, Surgeon, Great Ormond-street
Pratt, Mr. J. Surgeon, Malton
Ring, Mr. Surgeon, Conduit-street
Schumacher, Mr.
Stedman, S. Esq. Surgeon, Eltham
Turner, Mr. Surg. Ashton-under-Lyne
Warren, Dr. John, Boston
White, John, Esq. Consulting Physician to the Dispens. and House of Recovery, Paisley, President of the Paisley Medical Society
Wilde, Mr. M.R.C.S. &c. Mildenhall, Suffolk

ERRATUM.—In general List of Subscribers, Vol. II. *for* Cample, John, M.D. Surgeon, Ashton-under-line, *read* Campbell, M.D. Aston, U. L.

---

REMOVAL.

John Stevenson, Esq. from Great Russell Street to Margaret Street, Cavendish Square.

